# Metabolic and Neuroendocrine Responses to Intermittent Fasting in Obesity

**DOI:** 10.3390/medicina62020255

**Published:** 2026-01-25

**Authors:** Salvatore Allocca, Antonietta Monda, Maria Casillo, Fiorenzo Moscatelli, Marco La Marra, Vincenzo Monda, Girolamo Di Maio, Raffaele Ivan Cincione, Paride Vasco, Marcellino Monda, Rita Polito, Giovanni Messina, Antonietta Messina

**Affiliations:** 1 Unit of Dietetics and Sports Medicine, Section of Human Physiology, Department of Experimental Medicine, University of Campania “Luigi Vanvitelli”, 80138 Naples, Italy; salvatore.allocca01@unicampania.it (S.A.); maria.casillo@unicampania.it (M.C.); marco.lamarra@unicampania.it (M.L.M.); marcellino.monda@unicampania.it (M.M.); giovanni.messina@unicampania.it (G.M.); 2 Department of Human Science and Quality of Life Promotion, San Raffaele Telematic University, 00166 Rome, Italy; antonietta.monda@uniroma5.it; 3 Department of Education and Sport Sciences, Pegaso Telematic University, 80143 Naples, Italy; fiorenzo.moscatelli@unipegaso.it; 4 Department of Economics, Law, Cybersecurity and Sports Sciences, University of Naples “Parthenope”, 80131 Naples, Italy; vincenzo.monda@uniparthenope.it; 5 Department of Psychology and Health Sciences, Pegaso Telematic University, 80143 Naples, Italy; girolamo.diamio@unipegaso.it; 6 Department of Clinical and Experimental Medicine, University of Foggia, 71100 Foggia, Italy; ivan.cincione@unifg.it; 7 Department of Humanities, University of Foggia, 71100 Foggia, Italy; paride.vasco@unifg.it; 8 Department of Precision Medicine, University of Campania “Luigi Vanvitelli”, 80138 Naples, Italy; antonietta.messina@unicampania.it

**Keywords:** Mediterranean diet, circadian rhythm, obesity, inflammation, Orexin-A, metabolic flexibility

## Abstract

*Background and Objectives*: Intermittent fasting (IF) has emerged as a nutritional strategy capable of modulating circadian alignment, metabolic efficiency, and neuroendocrine regulation in individuals with obesity. Among the neurobiological mediators potentially involved, Orexin-A—a hypothalamic neuropeptide regulating arousal, appetite, and energy balance—may represent a key link between fasting patterns and metabolic homeostasis. This study aimed to evaluate the long-term metabolic and neuroendocrine effects of two intermittent fasting protocols, time-restricted feeding (16:8) and alternate-day fasting (5:2), compared with a hypocaloric Mediterranean diet used as a reference condition. *Materials and Methods*: Thirty adults with obesity (aged 20–40 years) were allocated to one of three dietary interventions—low-calorie Mediterranean diet, IF 16:8, or IF 5:2—based on habitual dietary patterns and followed prospectively for 12 months. Anthropometric parameters, metabolic indices, inflammatory markers (CRP, TNF-α, IL-6, IL-10), and circulating Orexin-A concentrations were assessed at baseline and at three-month intervals (T0–T3). *Results*: Both intermittent fasting protocols induced more rapid improvements in body mass index, adiposity, lipid profile, fasting glucose, and inflammatory markers compared with the Mediterranean diet. Among the IF strategies, the 16:8 regimen showed the most consistent and physiologically coherent pattern of adaptation, characterized by a progressive and sustained increase in Orexin-A levels. This response was strongly associated with enhanced metabolic flexibility, reduced systemic inflammation, and improved energy regulation over time. In contrast, the 5:2 protocol produced more variable metabolic and neuroendocrine responses, likely due to alternating cycles of marked caloric restriction and compensatory intake. *Conclusions*: Intermittent fasting, particularly the 16:8 time-restricted feeding protocol, appears to be an effective and sustainable chrononutritional strategy for obesity management. By reinforcing circadian organization, improving inflammatory balance, and activating orexinergic pathways, the 16:8 model emerges as a promising intervention to address key metabolic and neuroendocrine dysfunctions associated with obesity.

## 1. Introduction

Obesity is now widely understood as a complex multisystem disorder characterized not only by excessive energy storage but also by profound alterations in circadian organization, inflammatory tone, and neuroendocrine regulation [1]. Traditional models that framed obesity primarily as an imbalance between caloric intake and expenditure have been increasingly challenged by evidence demonstrating that biological timing plays a crucial role in metabolic health. Daily oscillations in feeding–fasting cycles, hormonal secretion, sleep–wake behavior, and thermogenesis are coordinated by an intricate network of central and peripheral clocks. When these rhythms are disrupted—whether through irregular meal timing, prolonged evening eating, poor sleep quality, or hypothalamic dysregulation—metabolic flexibility decreases, glucose tolerance worsens, and systemic inflammation increases, creating a physiological environment conducive to weight gain and metabolic disease [2,3,4]. Modern lifestyles have intensified these circadian disturbances. Erratic eating patterns, increased light exposure during nighttime hours, reduced physical activity, and chronic stress all contribute to a persistent misalignment between internal metabolic rhythms and external behavioral cues. This desynchronization alters mitochondrial efficiency, impairs lipid handling, and disrupts hormones central to energy balance, including insulin, cortisol, leptin, ghrelin, and the orexin neuropeptides [5]. As a result, individuals with obesity frequently exhibit a metabolic profile marked by inefficient substrate utilization, reduced energy expenditure, and heightened inflammatory signaling. In recent years, intermittent fasting (IF) has gained considerable attention as a dietary strategy capable of directly targeting these underlying mechanisms. Unlike traditional calorie-restricted diets that focus solely on the quantity of food consumed, IF protocols modify the timing of food intake, thereby engaging pathways linked to circadian biology. Approaches such as time-restricted feeding (e.g., 16:8 fasting) and alternate-day fasting (5:2) have been shown to influence a wide range of metabolic processes, including insulin sensitivity, adipose tissue remodeling, oxidative stress reduction, and autophagy regulation. By structuring prolonged fasting windows, IF may help recalibrate disrupted circadian rhythms and promote metabolic conditions that favor fat oxidation and improved glycemic control [6,7,8]. The neuroendocrine dimension of intermittent fasting has also become a compelling area of investigation. Of particular interest is Orexin-A, a hypothalamic neuropeptide that plays a central role in wakefulness regulation, spontaneous physical activity, appetite modulation, and energy expenditure. Lower orexinergic tone—frequently observed in individuals with obesity—has been associated with reduced metabolic efficiency, diminished thermogenesis, and increased susceptibility to weight gain [9,10]. Because Orexin-A is acutely responsive to feeding patterns, inflammatory signals, and energetic state, it represents a promising biomarker for understanding the neuroendocrine adaptations elicited by intermittent fasting. However, despite growing recognition of its physiological relevance, the long-term effects of IF on orexinergic activation remain insufficiently characterized in human populations [11,12]. Although alternative nutritional models—such as the Mediterranean Diet—have been explored for their anti-inflammatory and metabolic benefits, intermittent fasting distinguishes itself by its ability to modulate feeding time, circadian alignment, and neuroendocrine responses simultaneously. What remains unclear is whether different IF regimens exert comparable or distinct effects on key metabolic and hormonal pathways over prolonged periods, particularly in individuals with obesity whose circadian and neuroendocrine systems are already compromised [13,14,15]. Considering these gaps, the present study aims to investigate the metabolic and neuroendocrine responses to two widely adopted intermittent fasting protocols—a 16:8 time-restricted feeding schedule and a 5:2 alternate-day fasting model—over a 12-month period in individuals with obesity. By examining changes in body composition, metabolic biomarkers, inflammatory mediators, and Orexin-A levels, this work seeks to clarify the extent to which intermittent fasting influences neuroendocrine function and metabolic remodeling. Understanding these relationships may help identify fasting-based dietary strategies capable of restoring metabolic efficiency, reducing inflammation, and supporting circadian realignment in obesity.

## 2. Materials and Methods

### 2.1. Study Population

A total of thirty individuals with obesity (15 men and 15 women), aged 20–40 years, were enrolled from the Dietetics, Sports Medicine, and Psychophysical Well-Being Unit of the University Hospital “Luigi Vanvitelli” (University of Campania), under the supervision of Professor Marcellino Monda. After screening for eligibility, participants were categorized into three dietary groups (*n* = 10 per group) based on their habitual dietary patterns and monitored for 12 months. Prior to inclusion, all volunteers signed written informed consent forms. The entire study adhered to the principles of the Declaration of Helsinki and received approval from the Ethics Committee of the University of Campania “Luigi Vanvitelli” (protocol 0003232/1, issued on 1 February 2023).

### 2.2. Study Design

This study was conducted as a 12-month, non-interventional, observational comparison of three commonly adopted dietary approaches used in routine nutritional counseling for obesity: time-restricted feeding (16:8) and alternate-day fasting (5:2), compared with a traditional hypocaloric Mediterranean Diet After group selection, individuals were monitored from baseline (T0) to 12 months (T3) with repeated assessments of metabolic and hormonal parameters. No randomization, allocation procedures, or concealed assignment were used, as the study did not seek to test a therapeutic intervention but rather to observe naturally occurring differences among participants following dietary models already established in clinical practice. The research team did not alter participants’ usual care, did not prescribe interventions beyond standard dietary advice, and did not introduce any experimental treatment. All procedures adhered to a non-interventional design, focusing solely on longitudinal monitoring of outcomes.

### 2.3. Dietary Interventions

Three nutritional strategies were adopted in this study to evaluate their impact on metabolic function, inflammatory activity, and the prevention of obesity-related disturbances. The first approach, used as the reference group, consisted of a low-calorie Mediterranean Diet. Participants assigned to this regimen followed an energy-restricted plan providing 1200 kcal per day for women and 1500 kcal per day for men. The distribution of macronutrients was balanced to include approximately 55% of total calories from carbohydrates, 25% from fats, and 20% from proteins. Participants assigned to the Mediterranean diet consumed three main meals per day (breakfast, lunch, and dinner), with breakfast typically between 7:00 and 8:00 a.m., lunch between 1:00 and 2:00 p.m., and dinner between 7:00 and 8:00 p.m., reflecting conventional Mediterranean eating patterns. This nutritional model was selected because it reflects established clinical guidelines for weight management and therefore served as the standard comparator for the other interventions. The second intervention was the Intermittent Fasting 16:8 protocol. In this regimen, participants abstained from food for 16 consecutive hours each day and consumed all meals within a fixed 8 h eating window, typically between 12:00 p.m. and 8:00 p.m. Although the total daily caloric intake was comparable to that prescribed for the Mediterranean Diet group, it was confined entirely to this restricted period. During the eating window, the macronutrient composition was designed to provide roughly 40–45% of calories from carbohydrates, 30–35% from fats—primarily unsaturated sources—and 20–25% from proteins. Throughout the fasting phase, individuals were permitted to consume water, unsweetened tea, and black coffee. This structured alternation between prolonged fasting and restricted feeding is known to promote metabolic adaptability, facilitating increased fat oxidation and improvements in insulin sensitivity.

The third strategy implemented was the Intermittent Fasting 5:2 model. Participants assigned to this group followed two non-consecutive low-intake days each week, such as Monday and Thursday, while eating ad libitum on the remaining five days. On fasting days, caloric intake was limited to approximately 20–25% of individual daily requirements, corresponding to around 400–500 kcal. The macronutrient distribution on these days consisted of 30% carbohydrates, 45% fats—mainly omega-3 and other unsaturated fatty acids—and 25% proteins. Foods typically recommended included lean protein sources, vegetables, low-glycemic fruits, and modest quantities of healthy fats. This intermittent restriction pattern mimics the effects of continuous caloric reduction while allowing greater dietary flexibility, a characteristic that has been associated with enhanced adherence and measurable benefits in terms of weight loss and metabolic regulation. For the 16:8 protocol, total daily caloric intake was designed to be comparable to that of the Mediterranean diet group, with energy consumption concentrated within the 8 h eating window. For the 5:2 protocol, caloric comparability refers to average weekly energy intake rather than identical daily intake, as caloric restriction was applied only on two non-consecutive fasting days per week. Dietary adherence was monitored through weekly diet logs and structured dietary recalls at each visit. Trends in body composition (BIA) and metabolic parameters were also used as indirect adherence indicators.

### 2.4. Integrated Evaluation of Anthropometric, Metabolic, and Inflammatory Parameters

Evaluations were conducted at baseline (T0) and subsequently at three-month intervals (T1, T2, and T3), covering the entire 12-month intervention period. Anthropometric measurements were obtained using standardized procedures. Body height was measured to the nearest 0.1 cm using a wall-mounted stadiometer (Seca, Hamburg, Germany), and body weight was recorded to the nearest 0.1 kg using a calibrated digital scale (Seca, Hamburg, Germany). Body Mass Index (BMI) was calculated as body weight divided by height squared (kg/m^2^). Body composition was assessed by multifrequency bioelectrical impedance analysis (BIA) using a validated analyzer (BIA 101 Anniversary, Akern Srl, Florence, Italy). All measurements were performed under controlled conditions: participants were instructed to fast for at least 8 h, empty their bladder immediately before testing, avoid alcohol consumption for 24 h, and refrain from strenuous physical activity during the 24 h preceding the assessment. Measurements were carried out with participants in a supine position after a 10 min rest period. The BIA provided estimates of fat mass, fat-free mass, total body water, and the relative distribution of intracellular and extracellular water compartments. Resting energy metabolism was indirectly estimated using predictive equations based on fat-free mass values derived from the bioimpedance analysis, allowing calculation of individual daily energy expenditure. Venous blood samples were collected in the morning (between 08:00 and 09:00 a.m.) following an overnight fast of at least 8 h. Blood was drawn into EDTA-coated tubes and centrifuged at 3000 rpm for 15 min at 4 °C. Plasma was separated, aliquoted, and stored at −80 °C until analysis. Standard biochemical parameters, including fasting glucose, total cholesterol, high-density lipoprotein cholesterol (HDL-C), low-density lipoprotein cholesterol (LDL-C), and triglycerides, were measured using automated enzymatic methods at a certified clinical laboratory. Plasma inflammatory and anti-inflammatory markers were quantified to further characterize systemic inflammatory status. Concentrations of C-reactive protein (CRP), tumor necrosis factor-α (TNF-α), interleukin-6 (IL-6), and interleukin-10 (IL-10) were measured using commercially available enzyme-linked immunosorbent assay (ELISA) kits (R&D Systems, Minneapolis, MN, USA; Thermo Fisher Scientific, Waltham, MA, USA), according to the manufacturers’ instructions. Briefly, plasma samples and standards were added in duplicate to microplate wells pre-coated with specific capture antibodies and incubated under controlled conditions. After washing to remove unbound components, enzyme-conjugated secondary antibodies were applied, followed by the addition of tetramethylbenzidine (TMB) substrate to initiate the chromogenic reaction. The reaction was stopped using a dedicated stop solution, and absorbance was read at 450 nm using a calibrated microplate reader (Bio-Rad iMark, Hercules, CA, USA). Analyte concentrations were calculated from standard calibration curves generated for each assay.

All participants, regardless of dietary assignment, underwent the same assessment schedule, with measurements collected at baseline and subsequently every three months for the entire 12-month study period. This systematic monitoring allowed for a comprehensive evaluation of metabolic, inflammatory, and body-composition changes over time. Assessments were conducted at baseline (T0) and after 3 months (T1), 6 months (T2), and 12 months (T3).

### 2.5. Statistical Analysis

Statistical analyses were performed using GraphPad Prism (version 10.6.0 for Windows/Mac). All data are presented as mean ± standard deviation (SD) for each dietary group at each assessment time point (T0–T3).

To evaluate changes over time within groups and differences between dietary interventions, a repeated-measures analysis of variance (ANOVA) was applied. The model included the following:

(i) The main effect of time (temporal changes across assessment points);
(ii) The main effect of dietary group (Mediterranean Diet, IF 16:8, IF 5:2);
(iii) The interaction term time × group, assessing differences in temporal response patterns among interventions.

A two-sided significance level of α = 0.05 was adopted. When a significant main effect or interaction was detected (*p* < 0.05), the null hypothesis of equal means was rejected. For each outcome variable (BMI, fat mass, lean mass, total cholesterol, fasting glucose, and orexin-A), F-statistics, corresponding *p*-values, and coefficients of determination (R^2^) were reported.

Post hoc comparisons were performed using Bonferroni-adjusted tests to control for multiple comparisons. Specifically, post hoc analyses were conducted:

(i) Within each dietary group, comparing each follow-up time point (T1–T3) to baseline (T0);
(ii) Between dietary groups at corresponding time points.

Levels of statistical significance are reported as * *p* < 0.05; ** *p* < 0.01; *** *p* < 0.001.

## 3. Results

### 3.1. Evaluation of Anthropometric, Metabolic, and Hormonal Parameters

To assess the effects of the different dietary regimens—low-calorie Mediterranean diet, intermittent fasting (IF 16:8), and alternate-day fasting (5:2)—the main anthropometric, metabolic, and hormonal parameters were monitored throughout the study period at four time points (T0, T1, T2, and T3).

Specifically, the following variables were analyzed:

- Body Mass Index (BMI);
- Fat mass and lean mass percentage;
- Total cholesterol levels;
- Fasting blood glucose;
- Plasma Orexin-A concentrations.

These parameters were selected to comprehensively evaluate changes in body composition, metabolic efficiency, and hormonal adaptations associated with each dietary intervention over time.

Statistical analysis of body mass index (BMI) data in the three experimental groups showed significant differences over time. Repeated-measures ANOVA revealed a statistically significant main effect between groups (F = 7.602; *p* = 0.0005), confirming the presence of significant variations in BMI as a function of the type of dietary intervention. The coefficient of determination (R^2^ = 0.3878) indicates that approximately 39% of the observed variability in BMI values is explained by the model.

**Figure 1:**
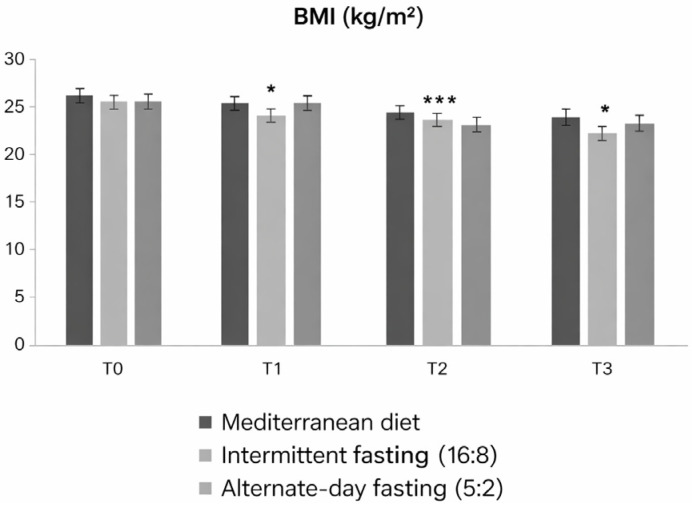
Body mass index (BMI) trends in the three experimental groups (Low-calorie Mediterranean diet, Intermittent fasting (16:8), alternate-day fasting (5:2)) at different time points (T0, T1, T2, T3). Values are expressed as mean ± standard deviation. Bonferroni-adjusted post hoc comparisons: within-group vs. T0; between-group at each time point. Statistically significant differences between groups are indicated with asterisks (* *p* < 0.05; *** *p* < 0.001).

As shown in the graph, starting from T1, a progressive reduction in BMI is observed, more marked in subjects undergoing intermittent fasting, which represents the most effective dietary approach. Multiple comparisons highlighted significant differences between the intermittent fasting and alternate-day fasting (5:2) groups already at T1 (*p* < 0.01), with a further consolidation of the trend at T2 and T3 (*p* < 0.05; ** *p* < 0.001). The low-Calorie Mediterranean diet group also showed a reduction in BMI, albeit less pronounced, while alternate-day fasting (5:2) resulted in a more modest and less stable decline over time (Figure 1).

Analysis of percentage body fat showed significant differences between the three diets over time. ANOVA confirmed a statistically significant main effect (F = 7.726; *p* = 0.0004), with an R^2^ value of 0.3917, indicating that approximately 39% of the observed variability was explained by the model.

**Figure 2:**
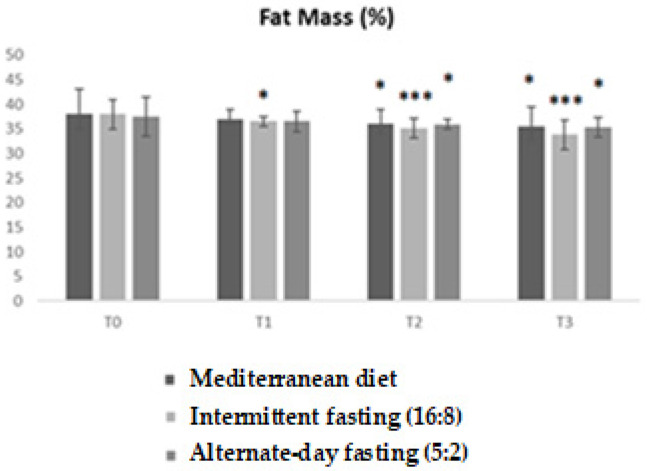
Trends in percentage body fat mass in the three experimental groups (low-calorie Mediterranean diet, intermittent fasting (16:8), and alternate-day fasting (5:2)) at different time points (T0, T1, T2, T3). Values are expressed as mean ± standard deviation. Bonferroni-adjusted post hoc comparisons: within-group vs. T0; between-group at each time point. Statistically significant differences between groups are indicated with asterisks (* *p* < 0.05; *** *p* < 0.001).

As illustrated in the graph, all experimental groups showed a progressive reduction in body fat from baseline (T0) to T3, but with varying degrees depending on the diet followed. In particular, the intermittent fasting group reported the most marked and consistent reduction, showing significant differences compared to the others already at T1 (*p* < 0.05) and with further strengthening of significance at T2 and T3 (* *p* < 0.01; ** *p* < 0.001). The low-calorie Mediterranean diet produced an intermediate reduction, while alternate-day fasting (5:2) resulted in less significant changes, remaining at higher values than intermittent fasting (Figure 2).

The analysis of percentage lean mass highlighted significant differences between the three diets over time. The ANOVA revealed a highly significant main effect (F = 11.54; *p* < 0.0001), with an R^2^ value of 0.4903, indicating that almost 50% of the observed variability is explained by the model.

**Figure 3:**
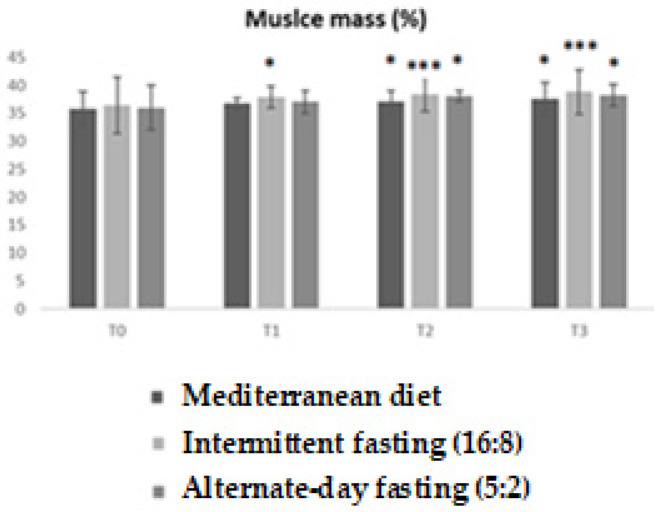
Trends in percentage lean mass in the three experimental groups (Low-calorie Mediterranean diet, Intermittent fasting (16:8), alternate-day fasting (5:2) diet) at different time points (T0, T1, T2, T3). Values are reported as mean ± standard deviation. Bonferroni-adjusted post hoc comparisons: within-group vs. T0; between-group at each time point. Statistically significant differences between groups are indicated by asterisks (* *p* < 0.05; *** *p* < 0.001).

The graph clearly shows that, while at baseline (T0) the three groups had comparable values, the differences progressively amplified over the course of the study. In particular, the intermittent fasting group showed a significant increase in lean mass already at T2 (*p* < 0.05; * *p* < 0.01), with further increases confirmed at T3 (** *p* < 0.001). The Low-calorie Mediterranean diet also showed an improvement, albeit to a lesser extent, while alternate-day fasting (5:2) did not produce equally marked changes (Figure 3).

Analysis of total cholesterol values revealed significant differences between the experimental groups over time. ANOVA showed a significant main effect (F = 5.955; *p* = 0.0021), with an R^2^ of 0.3317, indicating that approximately 33% of the observed variability is explained by the model.

**Figure 4:**
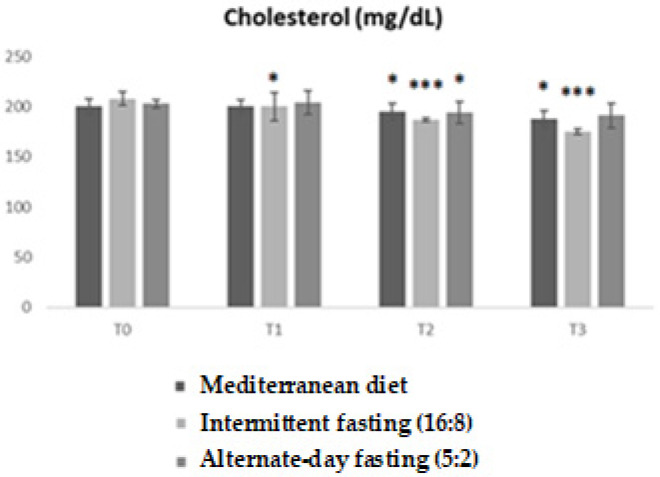
Trends in total cholesterol (mg/dL) in the three experimental groups (Low-calorie Mediterranean diet, Intermittent fasting (16:8), alternate-day fasting (5:2)) at different time points (T0, T1, T2, T3). Values are reported as mean ± standard deviation. Bonferroni-adjusted post hoc comparisons: within-group vs. T0; between-group at each time point. Statistically significant differences between groups are indicated by asterisks (* *p* < 0.05; *** *p* < 0.001).

The graph shows that, starting from T1, differential changes are observed between groups, with a more marked reduction in subjects undergoing intermittent fasting. This group showed significantly lower values than the others already at T1 (*p* < 0.05), with a progressive and significant reduction consolidated at T2 and T3 (* *p* < 0.05; ** *p* < 0.01; *** *p* < 0.001). The low-calorie Mediterranean diet induced a decrease in cholesterol, albeit more gradually, while alternate-day fasting (5:2) showed a less favorable trend, with more modest reductions (Figure 4).

Analysis of blood glucose values revealed significant differences between the three diet groups over time. ANOVA confirmed a significant main effect (F = 6.183; *p* = 0.0017), with an R^2^ of 0.3401, indicating that approximately 34% of the observed variability is explained by the model.

**Figure 5:**
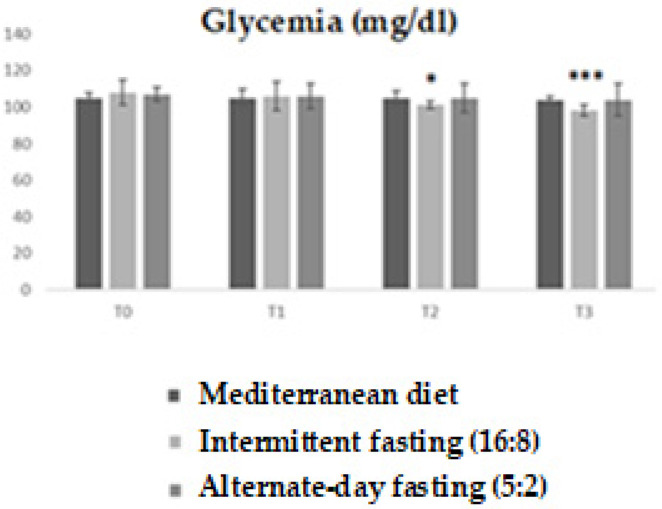
Blood glucose trends (mg/dL) in the three experimental groups (low-calorie Mediterranean diet, Intermittent fasting (16:8), alternate-day fasting (5:2)) at different time points (T0, T1, T2, T3). Values are reported as mean ± standard deviation. Bonferroni-adjusted post hoc comparisons: within-group vs. T0; between-group at each time point. Statistically significant differences between groups are indicated by asterisks (* *p* < 0.05; *** *p* < 0.001).

The graph shows that, starting from T2, subjects undergoing intermittent fasting showed a significant reduction in blood glucose levels compared to the other groups (*p* < 0.05), with a further decrease consolidated at T3 (* *p* < 0.05; ** *p* < 0.01; *** *p* < 0.001). The low-calorie Mediterranean diet produced a moderate and progressive decrease, while alternate-day fasting (5:2) showed a less favorable trend, with less consistent and statistically less significant reductions (Figure 5).

**Figure 6:**
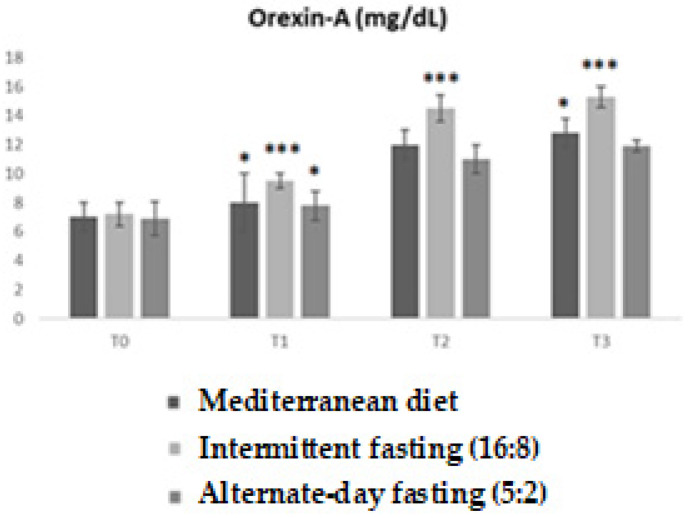
Trend of plasma Orexin-A levels (ng/mL) in the three experimental groups (Mediterranean diet, Intermittent fasting (16:8), alternate-day fasting (5:2)) at different time points (T0, T1, T2, T3). Values are expressed as mean ± standard deviation. Bonferroni-adjusted post hoc comparisons: within-group vs. T0; between-group at each time point. Statistically significant differences between groups are indicated with asterisks (* *p* < 0.05; *** *p* < 0.001).

The analysis of plasma Orexin-A levels revealed significant differences among the dietary groups over time. Repeated-measures ANOVA showed a highly significant main effect (F = 10.02; *p* < 0.0001), with an R^2^ value of 0.4551, indicating that approximately 46% of the observed variability was explained by the model. From the graph, it can be observed that, as early as T1, the IF 16:8 group exhibited a significant increase in Orexin-A levels compared with the other groups (*p* < 0.05). This effect became more pronounced at T2 and T3 (** *p* < 0.001), with Orexin-A values remaining consistently higher than those observed in the low-calorie Mediterranean diet and IF 5:2 groups. The IF 5:2 group, although showing a slight increase, did not reach comparable levels of change. The low-calorie Mediterranean diet group showed a moderate and steady increase, positioning itself between the two extremes (Figure 6).

### 3.2. Inflammatory Markers

The analysis of inflammatory and anti-inflammatory markers revealed different trends based on the diet followed.

Specifically, CRP, TNF-α, and IL-6 values progressively decreased in all groups, but to varying degrees. Intermittent fasting showed the most marked and statistically significant reduction starting at T2 (*p* < 0.01), with a further decline at T3. The low-calorie Mediterranean diet induced a more gradual decrease, still significant at T3 (*p* < 0.05), while intermittent fasting caused only small, non-statistically significant changes.

Conversely, levels of IL-10, an anti-inflammatory cytokine, showed a progressive increase, more pronounced in the intermittent fasting group (*p* < 0.01) than in the Low-Calorie Mediterranean diet (*p* < 0.05). In the intermittent fasting group, the increase was modest and non-significant.

Overall, the data confirm that intermittent fasting is the dietary approach with the most favorable impact on the cytokine profile, characterized by a marked reduction in pro-inflammatory cytokines and a simultaneous increase in anti-inflammatory ones as reported in Table 1 and Table 2.

Overall, the data show that all the dietary regimens examined had effects on the subjects’ metabolic status, albeit to varying degrees. The low-calorie Mediterranean diet showed a favorable and consistent, but less significant, effect, while alternate-day fasting (5:2) induced more modest and unstable reductions in the parameters analyzed.

In contrast, intermittent fasting emerged as the most effective and consistent diet. It resulted in a significant reduction in BMI and fat mass, accompanied by a progressive increase in lean mass. At the same time, it proved to be the most effective in improving lipid and glycemic profiles, with a marked reduction in total cholesterol and blood sugar levels already in the early phases of the study.

Therefore, the overall results clearly show that intermittent fasting is the diet with the most favorable and significant impact on the main anthropometric, metabolic, and hormonal parameters considered, proving superior to both the low-calorie Mediterranean diet and alternate-day fasting (5:2).

## 4. Discussion

The present study provides further evidence that intermittent fasting (IF) regimens can modulate metabolic and neuroendocrine pathways in individuals with obesity. Obesity is increasingly recognized as a condition characterized by circadian misalignment, systemic inflammation, and impaired neuroendocrine signaling; therefore, nutritional strategies that address not only caloric intake but also meal timing may contribute to the restoration of physiological rhythms essential for metabolic regulation. In this context, our findings indicate that although both the 16:8 and 5:2 intermittent fasting protocols produced beneficial metabolic and neuroendocrine effects, the 16:8 regimen consistently yielded more stable, coherent, and sustained improvements, underscoring the importance of daily fasting–feeding cycles in shaping metabolic adaptations [15,16].

Compared with the low-calorie Mediterranean diet, intermittent fasting was associated with more rapid early changes in body weight, fat mass, and glycemic parameters. These early responses likely reflect acute metabolic adaptations to fasting, including reductions in insulin levels, enhanced lipolysis, and activation of cellular stress-response pathways such as autophagy [17,18]. However, analysis of long-term trajectories revealed that the 16:8 protocol maintained these benefits more steadily and with fewer fluctuations than the 5:2 model. The regular daily fasting window characteristic of the 16:8 regimen may facilitate a more reliable transition between fed and fasted states, thereby enhancing metabolic flexibility and improving lipid oxidation capacity [19,20].

A key contribution of this study is the characterization of Orexin-A responses across the three dietary interventions. While both intermittent fasting regimens influenced orexinergic signaling, the 16:8 protocol was associated with a more progressive and sustained increase in Orexin-A levels. This pattern suggests that predictable daily fasting cycles may entrain hypothalamic circuits involved in energy regulation more effectively than intermittent caloric restriction [21]. In contrast, the 5:2 protocol elicited more irregular Orexin-A fluctuations, possibly reflecting alternating phases of marked energy restriction and compensatory feeding. Given the established role of Orexin-A in wakefulness, spontaneous physical activity, sympathetic activation, and metabolic efficiency, the more stable orexinergic response observed with the 16:8 regimen may reflect a neuroendocrine environment more favorable to sustained energy expenditure and substrate utilization [22].

Nevertheless, although significant associations were observed between Orexin-A levels, metabolic improvements, and inflammatory markers, these relationships should be interpreted with caution. Given the observational nature of the analyses, causality cannot be inferred. Changes in Orexin-A may reflect indirect effects related to weight loss, improved metabolic efficiency, or reduced inflammatory burden, rather than a direct consequence of fasting-induced neuroendocrine modulation [12,21].

Substantial interindividual variability was observed within each dietary group, particularly in the alternate-day fasting (5:2) protocol. This finding suggests that responsiveness to intermittent fasting may vary considerably among individuals and likely reflects differences in metabolic phenotype, behavioral adaptation, and neuroendocrine sensitivity [23]. In the 5:2 group, the lack of statistically significant differences at some intermediate time points appears to be driven primarily by increased variability rather than by an absence of biological effect, as mean values consistently trended toward improvement [24].

Inflammatory markers further support the relative advantages of the 16:8 fasting model. While both intermittent fasting protocols reduced systemic inflammation, decreases in CRP, IL-6, and TNF-α were more consistent and persistent in the 16:8 group. These findings reinforce the hypothesis that stable circadian reinforcement provided by daily fasting windows may more effectively counteract chronic low-grade inflammation. Considering that inflammatory pathways can suppress orexinergic neurons, the more pronounced anti-inflammatory profile associated with the 16:8 regimen may also have contributed to the more coherent increase in Orexin-A levels [23,24,25].

An additional advantage of the 16:8 regimen concerns adherence and long-term sustainability. Participants frequently reported that the predictability and moderate nature of daily time-restricted eating were easier to integrate into everyday life than the more restrictive and intermittent demands of the 5:2 protocol [26,27,28]. From a clinical perspective, this greater feasibility is particularly relevant, as long-term adherence is essential for maintaining metabolic improvements [29].

Although the Mediterranean diet served as a valuable comparator due to its well-established metabolic and anti-inflammatory benefits, it did not elicit the same rapid metabolic transitions, orexinergic modulation, or circadian reinforcement observed with intermittent fasting, particularly with the 16:8 model [30,31]. Taken together, these findings suggest that the 16:8 intermittent fasting regimen may represent a balanced strategy that combines early metabolic benefits with long-term stability and improved neuroendocrine coherence.

Limitations should be acknowledged. The relatively small sample size limits statistical power and generalizability. The absence of blinding and allocation concealment may introduce bias, and dietary adherence was not assessed using objective measures. Furthermore, potential confounding factors such as physical activity, sleep patterns, and chronotype—known modulators of circadian and orexinergic regulation—were not quantitatively assessed and may have contributed to the observed interindividual variability. Finally, the reliance on circulating biomarkers represents a surrogate approach to neuroendocrine and inflammatory activity rather than a direct mechanistic assessment.

## 5. Conclusions

Overall, this study indicates that intermittent fasting may improve metabolic health in obesity through a combination of metabolic adaptations and broader neuroendocrine changes, with potential involvement of the orexinergic system. Sustained improvements in body composition, inflammatory markers, and indices of metabolic flexibility highlight the capacity of fasting-based interventions to target pathways commonly dysregulated in obesity.

Among the fasting strategies examined, the 16:8 time-restricted feeding protocol consistently emerged as the most effective, producing changes that were not only more rapid but also more stable and physiologically coherent over the 12-month intervention. The regular fasting–feeding cycle characteristic of the 16:8 regimen may provide a favorable temporal structure for circadian alignment, support orexinergic signaling, and enhance substrate utilization. Although causality cannot be inferred, the progressive increase in Orexin-A levels observed with the 16:8 protocol is compatible with improved regulation of hypothalamic pathways involved in energy expenditure, arousal, and spontaneous physical activity, which are effects that appeared less consistent in the 5:2 model.

Importantly, the feasibility and sustainability of the 16:8 approach further strengthen its potential clinical relevance. Participants generally reported greater ease in adhering to daily time-restricted eating compared with the more restrictive and fluctuating nature of the 5:2 protocol, supporting the notion that effective obesity interventions must balance physiological efficacy with lifestyle compatibility.

Future studies should include larger randomized controlled trials incorporating objective measures of dietary adherence, physical activity, sleep behavior, and circadian rhythms. In addition, molecular-level investigations focusing on orexinergic signaling, gut–brain axis communication, and circadian clock gene regulation will be essential to clarify the mechanisms linking fasting patterns to neuroendocrine and metabolic adaptations. Long-term follow-up studies are also warranted to assess the durability of these effects beyond the intervention period and to evaluate whether combining time-restricted feeding with other evidence-based nutritional strategies may further enhance therapeutic outcomes.

## Figures and Tables

**Table 1:** Mean trend of inflammatory (CRP, TNF-α, IL-6) and anti-inflammatory (IL-10) markers in the three dietary groups (Mediterranean diet, Intermittent fasting (16:8), alternate-day fasting (5:2)) at different observation times (T0–T3).

Marker (Time)	Low-Calorie Mediterranean Diet	Intermittent Fasting (16:8)	Alternate-Day Fasting (5:2)
PCR (mg/L) T0	5.01	5.67	4.45
PCR (mg/L) T1	4.51	4.01	4.73
PCR (mg/L) T2	3.81	3.03	4.23
PCR (mg/L) T3	3.21	2.21	3.90
TNF-α (pg/mL) T0	8.01	7.69	8.34
TNF-α (pg/mL) T1	7.51	7.01	7.72
TNF-α (pg/mL) T2	6.92	5.81	7.05
TNF-α (pg/mL) T3	6.08	4.27	6.56
IL-6 (pg/mL) T0	4.56	4.53	5.57
IL-6 (pg/mL) T1	4.13	3.80	4.33
IL-6 (pg/mL) T2	3.71	3.04	4.08
IL-6 (pg/mL) T3	3.10	2.40	3.60
IL-10 (pg/mL) T0	1.22	1.01	1.11
IL-10 (pg/mL) T1	1.50	1.70	1.45
IL-10 (pg/mL) T2	1.91	2.32	1.63
IL-10 (pg/mL) T3	2.30	2.91	1.90

**Table 2:** Comparison of baseline (T0) and final (T3) values of inflammatory and anti-inflammatory markers in the three dietary groups, with indication of statistically significant differences (*p*-value) or notsignificant (n.s.).

Marker (Tempo)	Low-Calorie Mediterranean Diet	Intermittent Fasting (16:8)	Alternate-Day Fasting (5:2)	*p*-Value (T0 vs. T3)
PCR (mg/L) T0	5.01	5.67	4.45	–
PCR (mg/L) T3	3.21	2.21	3.90	<0.01 (DI), <0.05 (DM), n.s. (DC)
TNF-α (pg/mL) T0	8.01	7.69	8.34	–
TNF-α (pg/mL) T3	6.08	4.27	6.56	<0.01 (DI), <0.05 (DM), n.s. (DC)
IL-6 (pg/mL) T0	4.56	4.53	5.57	–
IL-6 (pg/mL) T3	3.10	2.40	3.60	<0.01 (DI), <0.05 (DM), n.s. (DC)
IL-10 (pg/mL) T0	1.22	1.01	1.11	–
IL-10 (pg/mL) T3	2.30	2.91	1.90	<0.01 (DI), <0.05 (DM), n.s. (DC)

## Data Availability

The raw data supporting the conclusions of this article will be made available by the authors on request.
